# Dr. Davis’ Institute

**Published:** 1862-11

**Authors:** 


### DR. DAVIS' INSTITUTE.

It will be observed by reference to our advertising sheet that Dr. Henry G. Davis has opened an Institution in New York for the treatment of diseases of the Joints, including dislocations and deformities. The well known reputation of Dr. Davis and his untiring efforts to advance and improve this branch of medical practice, will insure for him and his institution a favorable recommendation by all physicians who have a knowledge of the principles upon which many diseases of the joints are so successfully treated by extension and other mechanical means. Every one will see the advantages of a special home for such invalids, and few surgeons but will gladly concede that great improvements in treatment have been made in this department of surgery within the last few years. By properly applied mechanical treatment, results are constantly obtained which would be regarded as perfectly impossible by those not familiar with its benefits. We have no doubt Dr. Davis' Institution will fully sustain the claims it makes, and offer superior advantages for this class of invalids.

---

*Dr. Charles K. Winne*, U. S. A., son of Dr. Charles Winne of this City, has been appointed Medical Director, with the duty of inspector of five large hospitals, containing 1500 sick and wounded men. Dr. Winne, though yet a young man in the profession, has thus been promoted to one of the most responsible positions in the army, which shows an appreciation on the part of the Government of real merit. This position gives the Doctor a fine opportunity to practice surgery, his favorite department of the profession, where we have no doubt he will gain many fresh laurels, and not only command the respect of the profession, but make many friends by his gentlemanly and highly professional bearing.

